# Identifying High-Risk Factors of Depression in Middle-Aged Persons with a Novel Sons and Spouses Bayesian Network Model

**DOI:** 10.3390/healthcare8040562

**Published:** 2020-12-15

**Authors:** Francis Joseph Costello, Cheong Kim, Chang Min Kang, Kun Chang Lee

**Affiliations:** 1 SKK Business School, Sungkyunkwan University, Seoul 03063, Korea; f.costello@g.skku.edu (F.J.C.); saga@g.skku.edu (C.K.); 77aktrp3@gmail.com (C.M.K.); 2 Airports Council International (ACI) World, Montreal, QC H4Z 1G8, Canada; 3 Department of Health Sciences & Technology, Samsung Advanced Institute for Health Sciences & Technology (SAIHST), Sungkyunkwan University, Seoul 06355, Korea

**Keywords:** depression, health informatics, Bayesian network, genetic algorithm, machine learning, KNHANES

## Abstract

It has been reported repeatedly that depression in middle-aged people may cause serious ramifications in public health. However, previous studies on this important research topic have focused on utilizing either traditional statistical methods (i.e., logistic regressions) or black-or-gray artificial intelligence (AI) methods (i.e., neural network, Support Vector Machine (SVM), ensemble). Previous studies lack suggesting more decision-maker-friendly methods, which need to produce clear interpretable results with information on cause and effect. For the sake of improving the quality of decisions of healthcare decision-makers, public health issues require identification of cause and effect information for any type of strategic healthcare initiative. In this sense, this paper proposes a novel approach to identify the main causes of depression in middle-aged people in Korea. The proposed method is the Sons and Spouses Bayesian network model, which is an extended version of conventional TAN (Tree-Augmented Naive Bayesian Network). The target dataset is a longitudinal dataset employed from the Korea National Health and Nutrition Examination Survey (KNHANES) database with a sample size of 8580. After developing the proposed Sons and Spouses Bayesian network model, we found thirteen main causes leading to depression. Then, genetic optimization was executed to reveal the most probable cause of depression in middle-aged people that would provide practical implications to field practitioners. Therefore, our proposed method can help healthcare decision-makers comprehend changes in depression status by employing what-if queries towards a target individual.

## 1. Introduction

Depression is a common illness that affects a vast amount of the population [1]. Some estimates in western-based samples have depression levels as (potentially) high as half of the population, although these figures have been scrutinized [2]. Until now, it has been widely accepted that middle-aged adults are among the least depressed age group in society. Based on the maturity hypothesis, it is thought that middle-aged adults have greater self-esteem and positive self-image [3]. Thus, prior research has continuously shown a U-shaped distribution in the data with pre-30s and post-70s shown to be the most venerable [3]. Unfortunately, this does not mean that middle-aged adults are not at risk of depression. In fact, research suggests that depression in middle-aged adults can start around their 40s and continue in an upward trend [3].

As depression is not attributable to a small number of factors, trying to understand all of the potential causes can be a complex problem. For example, it has been widely shown that people who suffer from depression are more likely to engage in excessive smoking and drinking, creating a doubled-edged sword of harm towards one’s mental and physical health [4]. Further, trends in depression have been strongly linked to differences within gender [5], as well as socioeconomic and health-related reasons, such as obesity [6]. Moreover, depression has been shown to intrude on fundamental biological processes that are used for regulating certain bodily functions, such as sleep and appetite [7].

One common solution proposed for dealing with these issues is antidepressant medication. These drugs have been shown to be effective as quick fixes for people suffering from depression; however, they are yet to be proven as effective, long-lasting solutions [8]. Based on numbers provided by the American Psychological Association (APA) in 2017, 12.7% of the American population were actively taking antidepressants, with 16.6% of the population falling between the ages of 40 and 59 [9]. In South Korea, for example, short-/long-term use of antidepressants for the same age group was shown to be 21.25% [10]. Based on these figures, it could be suggested that depression is potentially more prevalent in middle ages compared to what was previously found [3].

As the use of antidepressant drugs in the middle age population is seen to be relatively high, in this paper we wanted to explore and propose the use of machine learning and artificial intelligence (AI) to identify depression risk factors. These days, deep-learning and logistical reasoning have dominated the conceptual frameworks within the field of artificial intelligence (AI). This has meant that less attention has been focused on the probabilistic theoretical paradigms. Due to real-world demands, probabilistic models are being chosen more often for tackling complex problems—this is especially true when compared to black box models, such as deep learning [11].

Given the complexity of understanding depression, there are valid grounds for a research agenda that implements health informatic techniques. Therefore, the goal of this research paper is to provide an alternative solution for understanding depression and exploring potential early warning signs that can guide preventative measures. Specifically, we explore depression-related factors using Bayesian statistics. This approach involves three stages, including feature selection, a supervised Sons and Spouses Bayesian Network (SS) [12], and genetic optimization through a genetic algorithm for understanding what solutions show the greatest risk to inducing depression. Although not common within the medical field, this approach has been successfully implemented in exploring other medical fields, including adolescent obesity [13], and thus we believe this methodology is well adapted for the current issue presented in this study.

Henceforth, we look to combine the use of probabilistic AI in the pursuit of analyzing the most probable risk factors on depression; therefore, this research is guided by the following research questions:

RQ_1_: based on a machine-learned probabilistic Bayesian Network, what are the most probable antecedents of depression within middle-aged people?

RQ_2_: through an AI-based genetic algorithm, what solutions show the greatest risk to inducing depression within middle-aged people?

## 2. Materials and Methods

### 2.1. Data Used for Experimentation

This study is an analysis using data from the seventh Korea National Health and Nutrition Examination Survey (KNHANES) (2016–2018), conducted by the Korea Centers for Disease Control and Prevention. Among the total samples of 24,269 in the seventh KNHANES database, this study was conducted on 10,120 people that fell into a middle-aged category, including people in their 30s, 40s, and 50s. After excluding 1540 people whose data had too many missing values, the final sample included 8580 people. This selection process was influenced by prior work that dealt with the topic of depression from the KNHANES public dataset [14].

Independent variables were divided into eight variables based on demographic characteristics and 18 variables based on health-related traits. First, gender, age, income (quartile), marital status, job status, drinking, smoking, and body mass index (BMI) were selected for the demographic characteristic variables. Variables related to health include subjective health conditions, doctor’s diagnosis for 13 diseases (hypertension, stroke, myocardial infarction, angina, arthritis, osteoporosis, pulmonary tuberculosis, asthma, diabetes, thyroid disease, breast cancer, cervical cancer, and thyroid cancer), oral examination, and values of glycated hemoglobin, hemoglobin, and high-sensitivity of the C-reactive protein.

The dependent variable was selected from the Euro quality of life questionnaire 5-dimensional classification (EQ-5D) developed by the EuroQol. In this study, the samples that showed little or high anxiety/depression were merged into one, representing depression is present. Detailed classifications are shown in Table A1.

### 2.2. Machine Learning Approach Based on Bayesian Statistics

In order to explore our first research question, we implemented a Bayesian Network (BN). A BN is a directed acyclic graph in which nodes represent domain variables, and arcs between nodes represent probabilistic dependencies. A BN is used to model data by computing the conditional probability of a given node based upon values assigned to other nodes within the data space [15]. In the distinct case, and provided that the probability of *B* is not equal to zero, Bayes’ theorem relates the conditional and marginal probabilities of events *A* and *B* through the following calculation [15]:

(1) $$Pr\left[A|B\right]=P\left(A\right)*\frac{Pr\left[B|A\right]}{Pr\left[B\right]}$$

The original proposition of Bayes’ theorem stated that the *P*(*A*) of the equation is the prior probability or the “unconditional” probability of *A*. In other words, this means that it does not take into consideration any of the information of *B*. Further, *B* does not have to emerge after the event *A*. Next, for a Bayes’ model to produce evidence of a probable outcome, it must calculate the joint probability distribution of a network. As a Bayes’ network can produce exponential amounts of probabilities depending on the data size, Bayesian networks use a localized conditional distribution that acts as a compressor in terms of calculations. This joint probability distribution then must be calculated in full. This is where the chain rule is used to analyze all of the joint probabilities of the network created. This is represented with the following equation [15]:

(2) $$P\left(x_{i},\ldots ,x_{n}\right)=\;\underset{i}{\prod }P\left(x_{i}|pa_{i}\right)\;$$

In order to learn the Bayes’ network, there are two competing methods: mutual information and the Kullback–Leibler (KL) Divergence metric. In this research, the KL Divergence was employed as this approach allows for the comparison of two probability distributions [16]. If we represent two probability distributions as *A* and *B*, then we can calculate the strength of the direct relationship between two of the nodes (variables):

(3) $$D_{KL}\left(A\left(X\right)\|B\left(X\right)\right)=\;\underset{X}{\sum }A\left(X\right)\log_{2}\frac{A\left(X\right)}{B\left(X\right)}$$

**Figure 1:**
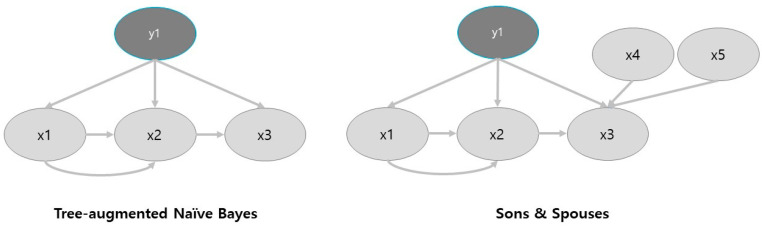
The main difference between the Tree Augmented Naïve Bayes model and the Sons and Spouses model. As can be seen, the Sons and Spouses model allows for relationships that are not directly linked to the target (father) node.

BNs have an advantage over other various predictive models, such as K-Nearest Neighbor (KNN) or Decision Trees (DT). This is because the Bayesian network structure represents the interrelations amongst attributes in the dataset explicitly [12]. This provides the benefit of being able to compute inference “omnidirectionally.” [17]. In this research, three Bayesian networks were considered: Naïve Bayes [15], Tree Augmented Naïve Bayes (TAN) [12], and Sons and Spouses (SS) [18]. First, Naïve Bayes was not used. This is because it is unable to filter out non-relevant nodes due to the fact it works in a Naïve fashion connecting all nodes to the parent node. Next, due to performance reasons, we selected to use the SS compared to the TAN model. SS is a variation of TAN; however, it allows for the possibility of relationships between the child nodes (sons), including two parent nodes (spouses; see Figure 1).

### 2.3. Artificial Intelligence Approach Based on a Genetic Algorithm

The purpose of this section is so that we can pursue research question two. Through the implementation of the previous approach using a BN, one can identify the relationships within data through machine learning. Despite this, it is hard to infer whether these relationships are causal. For this reason, an alternative or next step approach is necessary to further build upon the BN model that can be used as a predictor rather than observer. Based on the target optimization, we implemented the evolutionary search heuristic based on a Genetic algorithm (GA). This approach allows for a search of a “good explanation” based on a precise and concise exploration. In this setting, precise refers to removing the surprise value that is seen for explaining the relationships [19].

GA is a type of evolutionary algorithm designed to replicate real life. The search space is represented as a collection of individuals represented by the character strings, also known as chromosomes. Measured on an objective function, GAs attempt to find the best “genetic material” based on the given search space, otherwise known as the population [20]. GAs work in the following way: a population is chosen, and the quality of all data points are examined. Parents are selected from the population that then produce children. Newly spawned individuals in the population have a probability near zero so that they “mutate” into new potential individuals. Next, individuals are removed from the population based on a selection criterion until the population is the same size as before, known as one iteration or generation. In BN, children are selected based on two operators that help with the mutation, namely the crossover and mutation operators. Within the GA, mutation is responsible for exploring new states and avoiding local optima, whereas crossover looks to increase the average quality of the population. Coupled with a reduction factor, a GA can quickly find an optimum solution in a short amount of iterations [20].

For the purposes of our research, we use the generalized Bayes factor (*GBF*) as the measurement for selecting the best solutions provided by the GA. The *GBF* measure allows for the comparison of competing hypothesis that are presented to it, i.e., the best solutions are provided by the GA based on mutation and crossover of the BN. The *GBF* of a given explanation **x** out of all possible explanations $\bar{x}$ given the evidence **e** can be defined in the following way [19]:

(4) $$GBF\left(x;e\right)\equiv \frac{P\left(e|x\right)}{P\left(e|\bar{x}\right)}$$

The *GBF* has a unique property when it measures $P\left(e|\bar{x}\right)$. Specifically, when it measures two competing explanations ***x***, it has a symmetry in explanation. In other words, as one increases, the other decreases in a symmetric fashion. Given this fact, *GBF* essentially can be seen as the measure of change in the odds ratio of an explanation. However, as previously seen, BNs are based on multiple pieces of evidence, and thus implements the use of the chain rule. The same applies to *GBF* whereby a new explanation of **y** must be considered. Thus, the calculation is updated to [19]:

(5) $$GBF\left(y;e|x\right)\equiv \frac{P\left(e|y,x\right)}{P\left(e|\bar{y},x\right)}$$

and applied to the chain rule whereby $e_{1}$ represents the evidence to be true based on multiple pieces of evidence:

(6) $$GBF\left(x;e_{1},e_{2},\ldots ,e_{n}\right)=GBF\left(x;e_{1}\right)\overset{n}{\underset{i=2}{\prod }}GBF\left(x;e_{i}|e_{1},e_{2},\ldots ,e_{i-1}\right)$$

### 2.4. Benchmarked Classifiers

In order to gauge the performance of the selected method, benchmark classifiers were selected. This allowed for an easy comparison of the superior nature of Bayesian networks for the given target problem. Overall, nine commonly known algorithms that fall under various types of learning were selected, these included classical machine learning approaches, Bayesian methods, ensemble methods, and neural networks. The included algorithms were Tree Augmented Naïve Bayes (TAN) [12], Logistic Regression (LG) [15], Decision Tree (DT) [21], Neural Network (NN) [15], Support Vector Machine (SVM) [22], AdaBoost (ADA) [23], Bagging (BA) [24], Random Forest (RF) [25], and Random Subspace (RSS) [26].

## 3. Results

### 3.1. Feature Selection through Sons and Spouses Network Formulation

**Figure 2:**
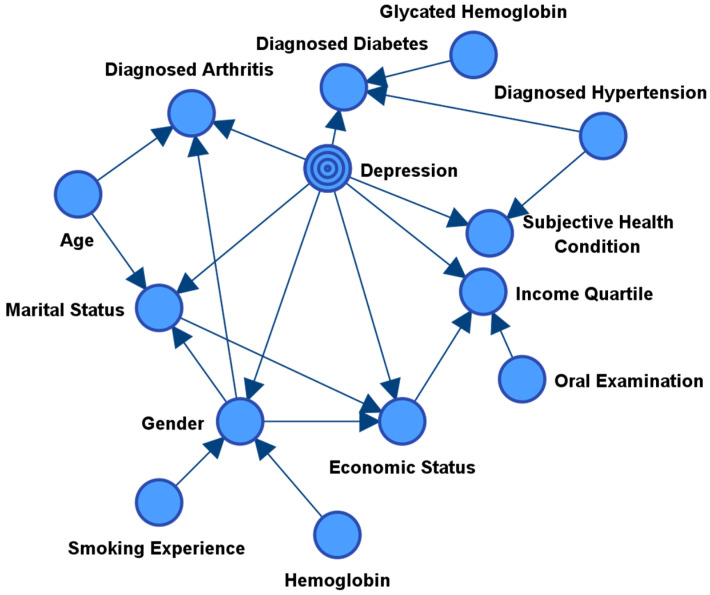
Variables selected with the Sons and Spouses machine learning algorithm and the connected nodes and arcs. As seen, marital status, economic status, income quartile, subjective health condition, gender, diagnosed with arthritis, and diagnosed with diabetes are direct child nodes of the target node depression.

The first task was to find the variables within the dataset that had the most information upon the target variable depression. This process not only helped to find the most significant variables as antecedents of depression, but it also helped to remove the redundant features from the network. The SS Bayes network was able to find 13 variables that had a direct or indirect relationship with the target variable (i.e., all the children and the parents of these children (spouses); see Figure 2). Furthermore, Table 1 shows all of the direct variables (children) that are having a direct relationship with the target node (see Table 1). According to this result, we can see that the top three variables influencing depression are found to be subjective health conditions, income quartile, and marital status. Based on this supervised machine-learning network, we next tested its predictive performance against the other classification models.

### 3.2. Benchmarking the Performance of the Obtained Sons and Spouses Model

In order to validate the use of Bayes statistics, we first compared the performance of the SS algorithm against well-known machine learning and ensemble learning techniques. Further, for all of the models, we implemented 10-fold cross-validation in order to validate the predictive results. As depression is a mental disease, it is not as common within the population and hence presents an imbalanced dataset. Thus, following prior research, we analyzed the performance based on accuracy, precision, and the F-Measure metric [13]. However, in medical classification tasks, the F-Measure is usually considered the best predictor. This is because the F-Measure considers both the recall and precision through a weighted average, meaning it punishes the classifier for false positives, an important feature when the dataset is sometimes about life and death situations [22].

Table 2 presents the results of the classification. As can be seen, the logistic classifier outperforms all of the other classifiers in terms of accuracy, with 93.32% accuracy. Compared with SS (93.04%), LOG was only a small margin greater in performance. For the AUC and F-Measure, the SS model outperforms all the other classifiers with 76.97% and 91.81%, respectively. Compared with LOG (AUC: 75.90%; F-Measure: 90.70%) and TAN (AUC: 75.20%; F-Measure: 92.59%), SS showed good performance, and thus justified our selection criteria. It is interesting to note that many of the classifiers, including popular models, such as SVM and NN, struggled to deal with the unbalanced data, and thus returned no F-Measure metric. This means that their prediction was unusable for comparison.

### 3.3. Identifying the Main Causes of Inducing and Reducing Depression with Genetic Optimization

Attempting to find the underlying causes and suggesting solutions can be a laborious task that is fraught with human error. For this reason, we next turned to AI. Based on a GA, we looked to analyze the probabilistic causes that are strong predictors of depression. As illustrated in Table 3, five models have been built showing a generalized Bayes factor of nearly 15 for each hypothesis: this means that the models provide strong evidence of having depression. Compared to other potential hypotheses found by the GA, these models have a greater probable cause of depression when all factors are considered in the BN. Specifically it presents three males and two females. All are in their 50s, except for one of the females. All are sufferers of arthritis and do not have current economic employment. This also means that they have low income. Diabetes was present in all males, yet none had a diagnosis of hypertension. Interestingly, all five solutions had varying conditions in their marital status and smoking experience. Interestingly, the data show that there is a potential link between being unmarried and smoking; however, this is not consistent. Lastly, three solutions showed very bad subjective health conditions, with one male having good, and another showing not bad. Lastly, as none of the authors are medical professionals, it is hard to analyze the remaining variables, however, the link between glycated hemoglobin and diabetes is well known, and thus higher levels, as seen in two males, may be a reflection of a twofold problem.

**Figure 3:**
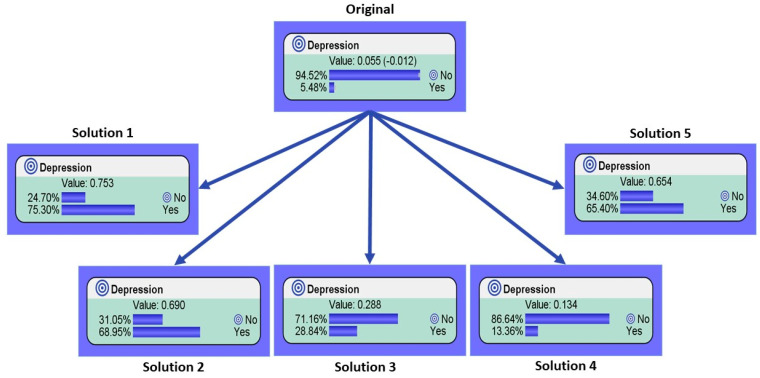
Based on the most probable causes found by the genetic optimization seen in Table 3, we explore the first three variables in Table 1, as well as gender and age, as these are non-subjective variables. The results show the difference from the original status of the population sampled (5.48%) and if a person was to have the values seen in these five variables suggested by the genetic algorithm.

As shown, the five solutions represent a hypothetical person. We most note however, the guiding principle of a best solution in this context is not for understanding the most probable cause of depression with 100% accuracy, it is more to show what factors need to be present at one time to show a greatly increased likelihood of depression. Although the GA has modelled five humans with 100% probability of having depression, reality tells us that these models are unattainable, yet useful in their application of identifying depression with just several of the factors. Hence, we show the benefits of this implemented system using a what-if analysis. For demonstration purposes, we explore the first three variables seen in Table 1 and the demographic factors of gender and age. As seen in Figure 3, compared with the original score of 5.48% depression amongst the whole sample, the genetic algorithm was able to find hypothetical patient conditions that would be high-risk for depression. As seen in outcomes 1, 2, and 5, the probability rate of having depression is above the threshold of 65%. In the case of outcomes 3 and 4, a rise in potential depression was seen to reach over 25% and 10%, respectively.

## 4. Discussion

Until now, research into middle-age depression has been limited to trend analysis [3]. Although the maturity hypothesis has been put forward to explain why this age group is at low risk, numbers indicating the use of depressive drugs within this age group suggest a different reality [9,10]. If we consider the pace of the modern economy and how quickly one’s life can change, for good and bad, analysis of the risk factors for depression in this age group has valid grounds for further research. Additionally, up until now, no research has focused on attempting to provide an early warning risk assessment model, in which practitioners can use to interpret depression in this age group. Thus, this research set out to tackle this problem using Bayesian statistics and AI. As AI techniques have developed in recent times, many options exist that can show strong predictive power. Unfortunately, in the case of methods such as deep learning, one cannot understand the decision-making process within the algorithm. Thus, it is hard to make grounded implications for medical purposes when multiple causes are at play [27].

To help overcome this problem, we employed the use of Bayesian statistics through a supervised network analysis. Specifically, we employed the Sons and Spouses (SS) network model in order to identify the antecedents that were having the greatest effect on depression. As Bayesian networks use the power of machine learning, there is no subjective insights that have led to its conclusions. As seen in Figure 1, the SS model provided seven direct sons and six spouses that were influencing depression. Furthermore, Table 1 showed the most important factors based on their relative binary mutual information, including: subjective health condition, income quartile, marital status, economic status, diagnosed arthritis, gender, and diagnosed diabetes. Next, due to the power of Bayesian networks and their interpretability, we employed a genetic algorithm (GA) in order to suggest the most probable causes of depression within our sample. By doing this, we were able to find variables that were suggested as high-risk causes of depression.

Compared with the 5.48% depression rate seen in the original data, based on the proposed solutions in this study, and the manipulation of five key variables’ values presented in the GA results, we found vastly different results. For example, in the first identified solution, the probability of depression within that hypothetical male example went up to 75.30% probability of being depressed. A similar effect was seen within two of the female solutions, whereby the proposed solutions showed an increase in the probability of depression to 68.95% and 65.40%, respectively. In addition, two other hypothetical male scenarios showed strong effects in terms of their probability of having depression rising to 28.84% and 13.36%, respectively.

The implications of this study can be seen through both an academic and practical viewpoint. First, academically, this research implemented a machine-learning Bayesian network approach, which was also seen in prior work within the medical field and public health [13]. However, prior work using Bayesian networks relies on subjective reasoning; thus, this is usually only accessible for medical practitioners. Therefore, to deal with this problem, we employed an AI-based genetic algorithm to help explore the target problem that did not require complex subjective knowledge. Based on this approach, an early warning risk application can be built that is induced with data-driven results opposed to one’s subjective approach. Practically, this has some key advantages. First, it makes this approach flexible. This is essential for depression research as cultural variations and local differences can be addressed in a quick and effective manner. Further, as this model is based on Bayesian statistics that use conditional probabilities, an evidence-based analysis can be made based on the features that a person has when they are being diagnosed. In other words, practitioners could implement this system with regionally localized data and find a number of solutions that predict the probability of depression. With this, they can then diagnose a patient’s probability of depression through updating the network with simple questions, such as age, gender, marital status, and subjective health conditions, to name a few.

## 5. Conclusions

This research paper looked to analyze depression within an understudied population: the middle-aged. By doing so, this research’s main goal was to create an approach based on a non-subjective machine learning and AI implemented system. The results showed that this methodology was effective in identifying highly probable causes of depression within middle-aged people. In addition, we were able to isolate the most probably causes of depression. Our results showed that this approach was able to effectively show probabilistic causes without the need for subjective knowledge. In implementing such a system, any practitioner could use this as an early warning risk application, by implementing a simple what-if analysis based on the probabilistic models found by a genetic optimized solution. This is because this method allows for an evidence-based analysis constructed on the features that a person has when they are being diagnosed. Thus, this could potentially help identify depression early on, potentially decreasing the long-term risk of severe depression in a patient.

This research has some flaws that need to be addressed. Firstly, the selection criteria for the data meant large amounts of people were removed due to missing data and variables that were too messy. Thus, there may have been some key samples removed in the preprocessing stage that could have had an effect on the overall outcome. However, as the conclusion in this research is more about the approach over the data implications, per se, this issue can be carefully explored with a more reliable survey. Moreover, questions over the use of the given models can be made. However, as there are endless numbers of models just within the Bayesian family, a decision had to be made on limiting this. Future research can explore the various models that exist in the Bayesian family, and compare and contrast their performance in this given problem.

## Figures and Tables

**Table 1:** Descriptive statistics from the Sons and Spouses network (target variable: depression).

Node	Relative Binary Mutual Information	Posterior Mean Value	Max Bayes Factor for No Depression	Min Bayes Factor for No Depression	Max Bayes Factor for Depression	Min Bayes Factor for Depression
**Subjective Health Condition**	9.74%	1.59	Very Good (1.0647)	Very Bad (0.6807)	Very Bad (0.6807)	Very Good (1.0647)
**Income Quartile**	2.12%	1.50	High (1.0266)	Low (0.9530)	Low (0.9530)	High (1.0266)
**Marital Status**	1.84%	2.15	Married Live Together (1.0127)	Divorced (0.9183)	Divorced (0.9183)	Married Live Together (1.0127)
**Economic Status**	1.78%	0.77	Yes (1.0150)	No (0.9537)	No (0.9537)	Yes (1.0150)
**Diagnosed Arthritis**	0.56%	0.05	No (1.0035)	Yes (0.9308)	Yes (0.9308)	No (1.0035)
**Gender**	0.39%	0.46	Male (1.0128)	Female (0.9893)	Female (0.9893)	Male (1.0128)
**Diagnosed Diabetes**	0.19	0.04	No (1.0018)	Yes (0.9579)	Yes (0.9579)	No (1.0018)

**Table 2:** Performance of the Sons and Spouses algorithm against common benchmark models.

	SS	TAN	LOG	SVM	NN	DT	ADA	BA	RSS	RF
**F-Measure**										
Total	91.81	91.00	90.70	N/A	90.00	N/A	N/A	90.50	N/A	90.20
**Accuracy**										
Total	93.04	92.59	93.39	93.32	91.31	93.32	93.32	93.26	93.32	92.97
**AUC**										
Total	76.97	75.20	75.90	50.00	65.40	49.80	73.30	74.20	73.90	71.30
**Precision**										
Total	93.14	90.01	91.10	N/A	89.00	N/A	N/A	90.30	N/A	88.70
**Recall**										
Total	90.60	92.60	93.40	93.30	91.30	93.30	93.30	90.50	93.30	93.30

SS (Sons and Spouses), TAN (Tree Aumented Naïve Bayes), LOG (Logarithm), NN (Neural Network), DT (Decision Tree), ADA (Adaboost), BA (Bagging), RSS (Random Subspace), RF (Random Forest).

**Table 3:** Variables selected based on the genetically induced most probable cause of depression.

Most Probable Cause of Depression								
	**Age**	**Diagnosed Arthritis**	**Diagnosed Diabetes**	**Diagnosed Hypertension**	**Economic Status**	**Gender**	**Glycated Hemoglobin**	**Hemoglobin**
**1**	50s	Yes	Yes	No	No	Male	≤8(3/4)	≤10(1/7)
**2**	40s	Yes	No	Yes	No	Female	≤8(3/4)	≤16(6/7)
**3**	50s	Yes	Yes	No	No	Male	>8(4/4)	≤10(1/7)
**4**	50s	Yes	Yes	No	No	Male	>8(4/4)	≤10(1/7)
**5**	50s	Yes	Yes	No	No	Female	≤6(2/4)	≤15(5/7)
	**Income Quartile**		**Marital Status**	**Oral Examination**	**Smoking Experience**	**Subjective Health Condition**	**Generalized Bayes Factor**	
**1**	Low		Unmarried	Yes	Less than 5 packs	Very Bad	14.9738	
**2**	Low		Divorced	Yes	Non-smoker	Very Bad	14.9738	
**3**	Low		Married Live Separated	Yes	Less than 5 packs	Not Bad	14.9738	
**4**	Low		Married Live Separated	Yes	Non-smoker	Good	14.9738	
**5**	Low		Unmarried	No	Less than 5 packs	Very Bad	14.9738	

## Appendix A

**Table A1:** Variables used for analysis extracted from the Korea National Health and Nutrition Examination Survey (KNHANES) dataset based in South Korea.

ID	Name	Classification
Sex^2^	Gender	1. Male 2. Female
age^2^	Age	29~38 (30 s) 39~48 (40 s) 40~58 (50 s)
incm	Income Quartile (individual)	1. Low 2. Middle low 3. Middle high 4. High
marri_2	Marital status	1. Married, live together 2. Married, live separated 3. Separated by death 4. Divorced 5. Unmarried
EC1_1	Current economic status	1. Yes (Employed) 2. No (Unemployed, economically inactive)
BD1_11	Drinking frequency in one year	0. N/A 1. I haven’t drunk at all in the last one year. 2. Less than once a month 3. About once a month 4. 2–4 times a month 5. Two or three times a week. 6. More than four times a week
BS1_1	Smoking experience	1. Less than 5 packs (100 pieces) 2. Five packs (100 pieces) or more 3. Non-smoker
HE_BMI	Body mass index	1. Under Weigh (less than 18.5) 2. Normal Weight (18.5~22.9) 3. Over-Weight (23~29.9) 4. Mild Obesity (30~34.9) 5. Obese (35~39.9) 6. Severe Obesity (more than 39.9)
D_1_1	Subjective health condition	1.Very good 2.Good 3.Normal 4.Bad 5.Very bad
DI1_dg	Doctor’s diagnosis status for hypertension	0. No 1. Yes
DI3_dg	Doctor’s diagnosis status for stroke	
DI5_dg	Doctor’s diagnosis status for myocardial infarction	
DI6_dg	Doctor’s diagnosis status for angina	
DM1_dg	Doctor’s diagnosis status for arthritis	
DM4_dg	Doctor’s diagnosis status for osteoporosis	
DJ2_dg	Doctor’s diagnosis status for pulmonary tuberculosis	
DJ4_dg	Doctor’s diagnosis status for asthma	
DE1_dg	Doctor’s diagnosis status for diabetes	
DE2_dg	Doctor’s diagnosis status for thyroid	
DC4_dg	Doctor’s diagnosis status for breast cancer	
DC5_dg	Doctor’s diagnosis status for cervical cancer	
DC7_dg	Doctor’s diagnosis status for thyroid cancer	
HE_HbA1c	Glycated hemoglobin	__%
HE_HB	hemoglobin	__g/dL
HE_hsCRP	High-sensitivity of C-reactive protein	__mg/L
OR1_2	Oral examination in the last year	0. No 1. Yes
LQ_5EQL	EuroQoL: Anxiety/Depression	1. I’m not anxious/depressed 2. I’m anxious/depressed
